# Astrocytes convert network excitation to tonic inhibition of neurons

**DOI:** 10.1186/1741-7007-10-26

**Published:** 2012-03-15

**Authors:** László Héja, Gabriella Nyitrai, Orsolya Kékesi, Árpád Dobolyi, Pál Szabó, Richárd Fiáth, István Ulbert, Borbála Pál-Szenthe, Miklós Palkovits, Julianna Kardos

**Affiliations:** 1 Department of Functional Pharmacology, Institute of Molecular Pharmacology, Research Centre for Natural Sciences, Hungarian Academy of Sciences, Pusztaszeri 59-67, 1025 Budapest, Hungary; 2 Laboratory of Neuromorphology and Neuroendocrinology, Semmelweis University and Hungarian Academy of Sciences, Tűzoltó 58, 1094 Budapest, Hungary; 3 Department of Biochemical Pharmacology, Institute of Molecular Pharmacology, Research Centre for Natural Sciences, Hungarian Academy of Sciences, Pusztaszeri 59-67, 1025 Budapest, Hungary; 4 Comparative Psychophysiology Group, Institute of Cognitive Neuroscience and Psychology, Research Centre for Natural Sciences, Hungarian Academy of Sciences, Victor Hugo 18-22, 1132 Budapest, Hungary; 5 Péter Pázmány Catholic University, Faculty of Information Technology, Práter 50A, 1083 Budapest, Hungary

## Abstract

**Background:**

Glutamate and γ-aminobutyric acid (GABA) transporters play important roles in balancing excitatory and inhibitory signals in the brain. Increasing evidence suggest that they may act concertedly to regulate extracellular levels of the neurotransmitters.

**Results:**

Here we present evidence that glutamate uptake-induced release of GABA from astrocytes has a direct impact on the excitability of pyramidal neurons in the hippocampus. We demonstrate that GABA, synthesized from the polyamine putrescine, is released from astrocytes by the reverse action of glial GABA transporter (GAT) subtypes GAT-2 or GAT-3. GABA release can be prevented by blocking glutamate uptake with the non-transportable inhibitor DHK, confirming that it is the glutamate transporter activity that triggers the reversal of GABA transporters, conceivably by elevating the intracellular Na^+ ^concentration in astrocytes. The released GABA significantly contributes to the tonic inhibition of neurons in a network activity-dependent manner. Blockade of the Glu/GABA exchange mechanism increases the duration of seizure-like events in the low-[Mg^2+^] *in vitro *model of epilepsy. Under *in vivo *conditions the increased GABA release modulates the power of gamma range oscillation in the CA1 region, suggesting that the Glu/GABA exchange mechanism is also functioning in the intact hippocampus under physiological conditions.

**Conclusions:**

The results suggest the existence of a novel molecular mechanism by which astrocytes transform glutamat*ergic *excitation into GABA*ergic *inhibition providing an adjustable, *in situ *negative feedback on the excitability of neurons.

## Background

Glial cells have long been considered to have only a supporting role in the central nervous system. Substantial advances in the past two decades, however, shed light on the various physiological functions they perform and led to the current view that they are active participants of the tripartite synapse [1,2], consisting of the presynaptic and postsynaptic neurons as well as the glial cells, in particular astrocytes. Several studies demonstrated the ability of astrocytes to sense, respond to and regulate neuronal function. Importantly, astrocytes possess the complete set of membrane proteins to detect γ-aminobutyric acid (GABA) and glutamate (Glu), the major inhibitory and excitatory neurotransmitters of the brain, respectively. They express GABA [3] and Glu [4] transporters, as well as ionotropic and metabotropic GABA [5-7] and Glu receptors [8]. Activation of these proteins initiates various pathways in the glial cells including Ca^2+^signalling [9,10], eventually leading to the release of GABA or Glu in either vesicular [11] or non-vesicular [12] manner.

Among the various mechanisms by which astrocytes control neuronal functions, regulation of tonic inhibition may play a major role. GABA*ergic *inhibition of neurons can be divided into phasic and tonic components. The phasic component is composed of discrete, transient inhibitory postsynaptic currents (IPSCs) corresponding to vesicular release events from the presynaptic axon terminal. These currents are mediated by low-affinity GABA_A _receptors in the synapse. In contrast, the persistent tonic GABA*ergic *inhibition is mediated by high-affinity, slowly desensitizing, extrasynaptic GABA_A _receptors experiencing low concentrations of ambient GABA [13]. Tonic inhibition has recently been demonstrated to have paramount importance. By modulating the membrane conductance of the postsynaptic neurons, tonic inhibition greatly influences the excitability of single neurons as well as networks [14]. Moreover, tonic inhibition gains increasing attention in the treatment of pathophysiological conditions, like epilepsy [15] or stroke [16]. Despite its significance, however, the source of GABA that generates tonic current is still a matter of debate. Some groups claim that ambient GABA concentration is set almost exclusively by vesicular GABA escaping the synapse [17], while others argue that release from astrocytes constitutes a significant source for extrasynaptic GABA [18].

In addition to the well-known interplay between the GABA*ergic *and glutamat*ergic *systems at the cellular and sub-cellular levels [19-23], we previously demonstrated the existence of a direct interaction between GABA*ergic *and glutamat*ergic *neurotransmissions at the molecular level [24]. We showed that uptake of Glu triggered an elevation in the extracellular level of GABA both *in vitro *and *in vivo*. The direct coupling between excitatory and inhibitory neurotransmitter transporters was found to be independent of Glu receptor-mediated depolarization, external presence of Ca^2+ ^and glutamate decarboxylase activity. It was abolished in the presence of non-transportable blockers of either glial Glu or GABA transporters, suggesting that the concerted action of these transporters underlies the process [24].

In the present study, we explore the potential physiological and pathophysiological role of the Glu/GABA exchange process in freshly isolated hippocampal slices and in the hippocampus *in vivo*. We demonstrate that the GABA released from astrocytes in response to Glu uptake significantly contributes to the tonic inhibition of neurons during intense excitation. Moreover, the generated tonic inhibition emerges in line with the increasing network activity, providing a tuneable, *in situ *negative feedback. We also describe the molecular mechanism by which glutamat*ergic *neurotransmission is transformed into GABA*ergic *inhibition and identify the source of releasable astrocytic GABA. We show that the negative feedback control of astrocytes on neuronal excitability offers significant neuroprotection during seizure-like activity. The physiological importance of the Glu/GABA exchange mechanism is further substantiated by *in vivo *results, which are in accordance with our *in vitro *observations.

## Results

### Glial Glu and GABA transporters are colocalized

We previously demonstrated that Glu uptake evokes GABA release by reversal of glial GABA transporter (GAT) subtypes GAT-2 or GAT-3 in brain homogenates [24]. In the same study we also showed by costainings with the neuronal and glial markers NeuN, synapthophysin and GFAP that, in accordance with the literature [25], GAT-3 transporters are expressed mostly in astrocytes [24]. Furthermore, ultrastructural [25] and functional [26] data from other groups showed that GAT-3 is expressed on glial processes ensheathing excitatory synapses. Since the major glutamate transporters EAAT1 and EAAT2 are also localized to astrocytic end feet [27,28], it is a viable hypothesis that GAT-3 and EAATs are coexpressed on astrocytic end feet surrounding glutamat*ergic *synapses.

**Figure 1:**
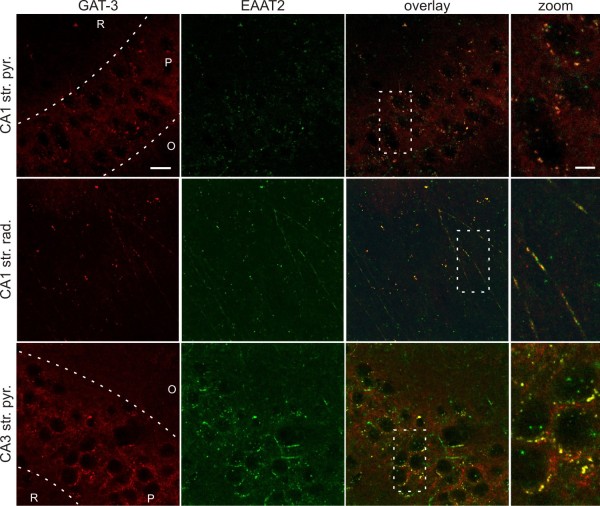
**Glial GABA and Glu transporters are colocalized**. GAT-3 (red) and EAAT2 (green) immunoreactive small puncta surround neuronal cell bodies and dendrites in the CA1 str. pyramidale (top), CA1 str. radiatum (middle) and CA3 str. pyramidale (bottom) regions. GAT-3 and EAAT2 immunoreactivites show almost complete colocalization (yellow) around the soma and on the dendrites of presumable pyramidal cells. Right: higher magnification of boxed region in merged image. R: str. radiatum, P: str. pyramidale, O: str. oriens. Scale bar = 50 μm (GAT-3, EAAT2 and overlay) or scale bar = 10 μM (zoom). All images are Z-projections of 6 optical sections taken at 2 μm distance.

To evaluate the possibility that GABA and Glu transporters colocalize in hippocampal slices, we performed GAT-3/EAAT2 double immunostaining. Both stainings showed punctate structures throughout the hippocampus. In accordance with data from other groups, EAAT2 immunoreactivity was most prominent in the dentate gyrus and in the pyramidal cell layer of the CA1 [27,28], while GAT-3 was mainly localized to the pyramidal layer of CA1 and CA3 [25]. Most importantly, the EAAT2 immunoreactivity was largely colocalized with GAT-3 in the pyramidal cell layer and stratum radiatum of both the CA1 and CA3 (Figure 1) region of the hippocampus. The immunoreactive puncta surrounded the cell bodies (Figure 1A, C) and dendrites (Figure 1B) of the glutamat*ergic *pyramidal neurons at presumed astrocytic end feet sites. To quantify the degree of colocalization, we determined the Pearson's coefficient (Rr) and the Mander's coefficient (R) [29]. The quantification showed high correlation between EAAT2 and GAT-3 immunoreactivity (Rr = 0.600, 0.516 and 0.566; R = 0.916, 0.893 and 0.764 for CA1 str. pyramidale, CA1 str. radiatum and CA3 str. pyramidale, respectively). The immunohistochemical analysis of the localization of GABA and Glu transporters, therefore, confirms the possibility of close, molecular-level interaction between EAAT2 and GAT-2/3 glial cells. Although a minor fraction of GAT-2/3 and EAAT2 may be expressed on neurons as well [30,31], their density in neurons is much lower than in astrocytes; therefore, they are unlikely to affect each other's activity even if they were colocalized.

### Glu uptake-induced GABA release through glial transporters contributes to tonic inhibition of neurons

Since the glial GAT-2/3 transporters are facing the extrasynaptic space instead of the synapse [26], the potential consequence of a transporter mediated glial GABA release is the activation of extrasynaptic GABA_A _receptors and the subsequent enhancement of tonic inhibition on neurons. To investigate the contribution of glial GATs to neuronal GABA*ergic *currents, we performed electrophysiological recordings from CA1 pyramidal cells of freshly isolated rat hippocampal slices under control conditions and during enhanced activity of the glutamat*ergic *synapses.

The impact of transporter mediated glial GABA release on neuronal tonic inhibition was assessed by blocking the glial GABA transporters with the GAT-2/3-specific, non-transportable inhibitor SNAP-5114 (100 μM, [9,32]) while measuring the GABA*ergic *holding current in CA1 pyramidal neurons that are extensively covered by a glial sheat [33]. We used CsMeSO_3_-based pipette solution to isolate GABA*ergic *(outward directed) currents in voltage clamped configuration by applying 0 mV holding potential to eliminate glutamat*ergic *currents. Synaptic currents were identified as GABA*ergic *inhibitory postsynaptic currents (IPSCs) and baseline currents were validated as a measure of tonic GABA*ergic *currents by adding the GABA_A _antagonist picrotoxin (100 μM) at the end of 43 of 154 experiments (*see *Methods). The GAT-2/3 mediated tonic current component was calculated as the change in the holding current in response to SNAP-5114 application.

**Figure 2:**
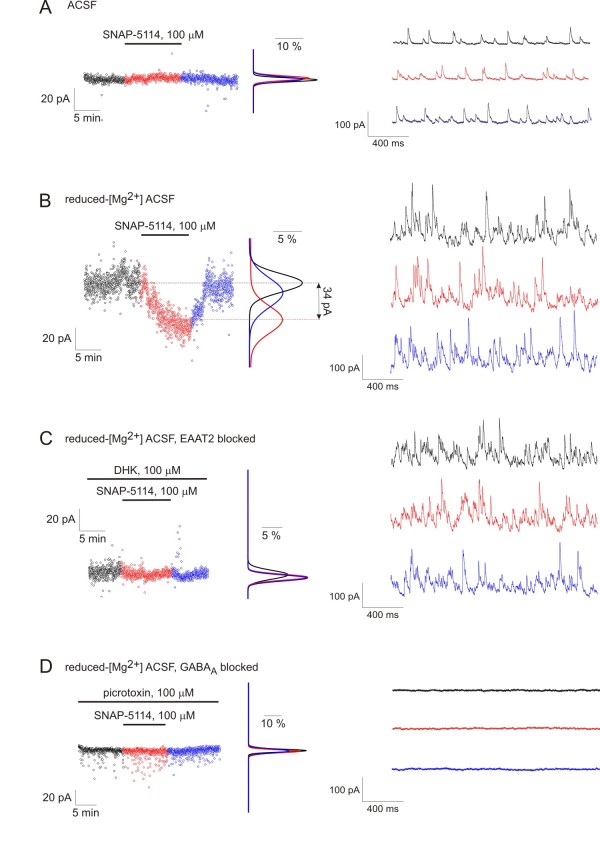
**Glu uptake-induced reversal of glial GABA transporters contributes to tonic inhibition of neurons during enhanced network activity**. Left, holding currents from representative experiments plotted at 1 s intervals during control condition (black), SNAP-5114 application (red) and washout (blue) with Gaussian fits to the histograms of the holding current recorded during each condition. *Right*, voltage clamp recording segments (V_h _= 0 mV) low-pass filtered at 1 kHz from the same experiments showed at left. **(A) **Effect of GAT-2/3 blockade by 100 μM SNAP-5114 on the holding current in [Mg^2+^] = 1,800 μM buffer. **(B) **Effect of GAT-2/3 blockade by 100 μM SNAP-5114 on the holding current in [Mg^2+^] = 10 μM buffer. **(C) **Effect of GAT-2/3 blockade by 100 μM SNAP-5114 on the holding current in [Mg^2+^] = 1 μM buffer in the presence of the non-transportable Glu uptake blocker DHK (100 μM). Average holding current: 88.5 ± 26.4 pA in the presence of 100 μM DHK vs. 93.6 ± 27.2 pA in the presence of 100 μM DHK and 100 μM SNAP-5114, *P *= 0.31, N = 8 cells/5 animals. **(D) **Effect of GAT-2/3 blockade by 100 μM SNAP-5114 on the holding current in [Mg^2+^] = 10 μM buffer in the presence of 100 μM picrotoxin. Average holding current: 9.3 ± 15.6 pA in control vs. 7.5 ± 12.7 pA in the presence of 100 μM SNAP-5114, *P *= 0.58, N = 4 cells/2 animals.

**Figure 3:**
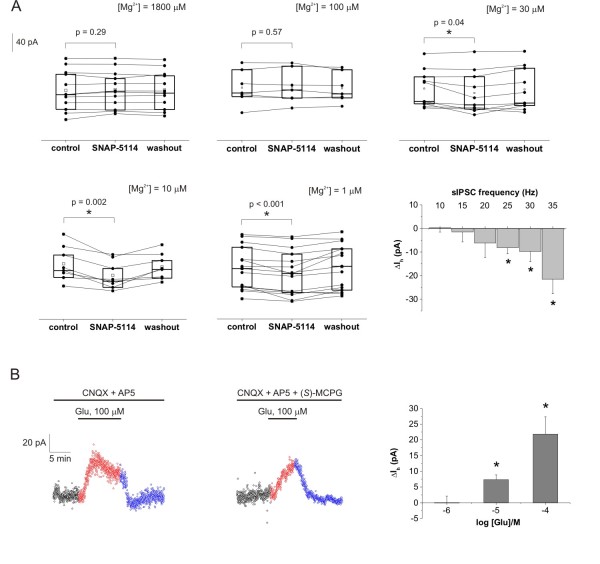
**Glial GABA transporter mediated tonic inhibition correlates with network activity**. **(A) **Box-chart representation of the average holding currents from individual experiments during control condition, SNAP-5114 application and washout. Box edges represent 25^th^, 50^th ^and 75^th ^percentile, open squares represent means, circles connected by lines represent paired individual baseline values. Average holding current in control: [Mg^2+^] = 1,800 μM: 78.9 ± 12.4 pA (N = 12 cells/2 animals); [Mg^2+^] = 100 μM: 63.0 ± 12.0 pA (N = 7 cells/3 animals); [Mg^2+^] = 30 μM: 155.0 ± 12.6 pA (N = 10 cells/3 animals); [Mg^2+^] = 10 μM: 121.2 ± 13.3 pA(N = 8 cells/4 animals); [Mg^2+^] = 1 μM: 88.8 ± 11.8 (N = 15 cells/9 animals); Bottom right*: *Average change in holding current in response to SNAP-5114 application as a function of the sIPSC frequency. Columns represent the means of holding current changes from all experiments having sIPSC frequency in the given 5 Hz wide frequency range during the control period. **(B) **Left*: *Holding currents during exogenous Glu application (100 μM, red) and washout (blue) in [Mg^2+^] = 10 μM buffer in the presence of Glu receptor antagonists CNQX (10 μM) and DL-AP5 (50 μM). Average holding current: 66.2 ± 23.5 pA in control vs. 93.0 ± 27.2 pA in the presence of 100 μM Glu, *P *< 0.001, N = 15 cells/8 animals. Middle*: *Holding currents during exogenous Glu application (100 μM, red) and washout (blue) in [Mg^2+^] = 10 μM buffer in the presence of Glu receptor antagonists CNQX (10 μM),DL-AP5 (50 μM) and (*S*)-MCPG (500 μM). Average holding current: 54.3 ± 17.4 pA in control vs. 69.1 ± 17.1 pA in the presence of 100 μM Glu, *P *= 0.01, N = 5 cells/4 animals. Right*: *Average change in holding current in response to increasing concentration (1 μM, 10 μM and 100 μM) of exogenous Glu in the presence of CNQX (10 μM) and DL-AP5 (50 μM). Asterisks represent significant change in holding current (*P *< 0.05).

Under control condition we did not observe significant change in the tonic current following blockade of GAT-2/3 transporters by 100 μM SNAP-5114 (Figures 2A and 3A). Local initiation of Glu release in the CA1 region by applying stimuli to the Schaffer collaterals by a bipolar Tungsten electrode also did not reveal a GAT-2/3 mediated tonic current component (average holding current: 74.9 ± 16.7 pA in control vs. 71.7 ± 17.1 pA in the presence of 100 μM SNAP-5114, *P *= 0.40, N = 8 cells/7 animals).

To investigate whether Glu uptake induced glial GAT reversal can occur under enhanced neuronal activity, we applied reduced-[Mg^2+^] buffers to remove the Mg^2+ ^blockade from NMDA receptors [34]. It is important to differentiate these reduced-[Mg^2+^] buffers from the low-[Mg^2+^] epilepsy model, used both in our lab [35] and by other groups [36]. The conditions applied in these experiments differ from seizure-generating conditions in two essential terms: (1) extracellular [K^+^] was not elevated in contrast to the slightly increased value (5 mM) used in seizure generation [37] and (2) we used 300 μm hippocampal slices in which the connectivity is less preserved than in the 400 μm slices usually utilized as an epilepsy model [35]. As a consequence, recurrent seizure-like events were observed in only 20% of the slices exposed to the [Mg^2+^] = 1 μM or [Mg^2+^] = 10 μM environment.

Application of [Mg^2+^] = 1 μM buffer resulted in a significant increase in both frequency (21.4 ± 2.2 Hz in control vs. 44.4 ± 2.5 Hz in reduced-[Mg^2+^], *P *< 0.001) and amplitude (28.6 ± 1.6 pA in control vs. 55.9 ± 6.9 pA in reduced-[Mg^2+^], *P *< 0.001) of spontaneous IPSCs (sIPSCs). Under these conditions, blockade of GAT-2/3 transporters significantly suppressed the tonic inhibition of CA1 pyramidal cells (Figures 2B and 3A), demonstrating that, during enhanced excitation, GABA release by reversal of GAT-2/3 significantly contributes to the tonic inhibition of neurons. This behavior is in sharp contrast to the direction of tonic current change we would expect from a GABA transporter working in the typical, outside-in direction, where the transporter blockade would lead to increased GABA*ergic *inhibition. SNAP-5114 application during combination of reduced-[Mg^2+^] condition with stimulation of Schaffer collaterals resulted in similar reduction of the tonic current (average holding current: 68.8 ± 18.7 pA in control vs. 49.3 ± 15.5 pA in the presence of 100 μM SNAP-5114, *P *= 0.04, N = 8 cells/7 animals). It is worth noting that neither the frequency (29.9 ± 1.5 Hz vs. 27.9 ± 1.4 Hz, *P *= 0.31), nor the peak amplitude (38.9 ± 4.4 pA vs. 33.3 ± 3.0 pA, *P *= 0.24) of the sIPSCs changed significantly during SNAP-5114 application compared to the preceding period, excluding the possibility that the decrease in the holding current is due to a change in IPSC kinetics.

To confirm that the GAT-2/3 mediated GABA release is directly evoked by Glu uptake, we investigated whether the SNAP-5114 sensitive tonic current component can be eliminated by blocking Glu transporters. We specifically blocked the glial EAAT2, the dominant Glu transporter subtype in the hippocampus [38]. If GAT reversal was evoked by glial EAAT activity, blockade of EAAT2 should eliminate the SNAP-5114-sensitive tonic current. On the contrary, if GABA release was triggered by Glu receptor activation, the higher extracellular Glu level due to EAAT blockade should result in enhanced SNAP-5114 sensitive tonic current component. In reduced-[Mg^2+^] buffer in the presence of the specific, non-transportable EAAT2 inhibitor DHK (100 μM), application of SNAP-5114 had no effect on the holding current (Figure 2C).

It is of note that the network activity in the presence of DHK was not significantly different from the reduced-[Mg^2+^] condition without DHK (frequency of IPSCs: 38.8 ± 1.9 Hz without DHK vs. 35.9 ± 4.7 Hz with DHK, *P *= 0.72), indicating that elimination of GAT-2/3 mediated tonic current cannot be attributed to desensitization of Glu receptors due to DHK blockade. We also excluded the possibility that the observed change in the holding current contributes to proteins other than the GABA_A _receptor. In the presence of the GABA_A _receptor antagonist picrotoxin (100 μM), blockade of GAT-2/3 by SNAP-5114 did not change the holding current (Figure 2D), confirming that GABA released from glial cells by GAT-2/3 reversal activates GABA_A _receptors.

Taken together these data demonstrate that during sustained excitation GABA released through GAT-2/3 in response to Glu transporter activation significantly contributes to tonic inhibition of CA1 pyramidal cells.

### GAT-2/3 mediated tonic component emerges gradually with increasing network activity

To investigate whether the GAT-2/3 mediated tonic component turns on beyond a threshold level or emerges gradually in line with the increasing network activity, we explored the effect of GAT-2/3 blockade on tonic GABA*ergic *currents in ACSF buffers with different levels of extracellular [Mg^2+^]. Extracellular [Mg^2+^] was varied in a wide range from the full blockade to the total unblock of the NMDA receptors [34] (Figure 3A). We showed that in the control buffer ([Mg^2+^] = 1,800 μM) there is no effect of GAT-2/3 blockade on the baseline current (Figures 2A and 3A). Applying 100 μM Mg^2+ ^that is still sufficient to block the NMDA receptors [34] also did not lead to GAT-2/3 reversal (Figure 3A). Partial unblock [34] of the NMDA receptors by 30 μM Mg^2+^, however, revealed the SNAP-5114-sensitive component in the GABA*ergic *tonic inhibition (Figure 3A). Further enhancement of the neuronal activity by applying 10 μM Mg^2+ ^or nominally Mg^2+^-free buffer (based on the Mg^2+ ^contamination of the Ca^2+ ^salts, we estimated the Mg^2+ ^concentration of this buffer to be approximately 1 μM) led to a robust increase in the contribution of GAT-2/3 reversal to the tonic current (Figure 3A).

To specifically measure the GABA*ergic *tonic current, we used CsMeSO_3 _based pipette solution at 0 mV holding potential, the reversal potential of Glu receptors. Therefore, we could not simultaneously record excitatory postsynaptic potentials and use these values to correlate network activity with the GAT-2/3 mediated tonic current component. To circumvent this limitation, we utilized the sIPSC frequency as readout of the network activity. Although this rough estimation of the network activity may not be ideal, the frequency of sIPSCs, indeed, significantly increased in the reduced-[Mg^2+^] ACSF (21.4 ± 2.2 Hz in control vs. 44.4 ± 2.5 Hz in [Mg^2+^] = 10 μM, *P *< 0.001), suggesting the reliability of this assumption. The GAT-2/3 mediated tonic component as a function of the sIPSC frequency (Figure 3A, bottom right) clearly shows that the tonic inhibition offered by astrocytes is proportional to the network activity.

### Tonic inhibition can be increased by exogenous Glu

We also assessed the level of excitatory drive required to induce the astrocytic GABA release by stimulating EAAT activity with different concentrations of exogenous Glu. Hippocampal slices were superfused with [Mg^2+^] = 10 μM buffer. Fifteen minutes prior to Glu application, the AMPA and NMDA receptors were blocked by antagonists CNQX (10 μM) and DL-AP5 (50 μM), respectively. Under these conditions, application of 1 μM Glu did not change the tonic GABA*ergic *current (Figure 3B, right). However, 10 μM Glu significantly increased the tonic inhibitory current (Figure 3B, right) without altering the synaptic sIPSC parameters (27.1 ± 2.3 Hz in control vs. 27.8 ± 2.3 Hz in the presence of 10 μM Glu, *P *= 0.81). Further increase of the tonic inhibition was observed in the presence of 100 μM Glu (Figure 3B, left). The unaffected sIPSC parameters indicate that most of the receptor-mediated actions of Glu were indeed blocked by the applied antagonists. This was further confirmed by the addition of the broad spectrum metabotropic Glu receptor antagonist (*S*)-MCPG to the buffer. 100 μM Glu was still able to significantly increase the holding current in the presence of 10 μM CNQX, 50 μM DL-AP5 and 500 μM (*S*)-MCPG (Figure 3B, middle). Although the increase in the holding current was slightly smaller in the presence of the metabotropic Glu receptor antagonist (*S*)-MCPG, suggesting the involvement of glutamat*ergic *activation of GABA*ergic *interneurons, the majority of the tonic inhibition increase is clearly attributed to Glu transporter activation. Additionally, blockade of the GABA release by the EAAT2-specific inhibitor DHK (Figure 2C) also supports the notion that Glu receptors do not considerably contribute to the increase in tonic inhibition, since any Glu receptor-mediated effect should have been intensified when Glu uptake was blocked. Altogether, these data demonstrate that GABA release from astrocytes can be directly evoked by physiologically relevant concentration of Glu through the activation of glial Glu transporters.

It is worth noting that under control conditions ([Mg^2+^] = 1,800 μM) we did not observe the enhancement of tonic inhibition following application of Glu up to 100 μM either in the presence or in the absence of Glu receptor antagonists CNQX (10 μM) and DL-AP5 (50 μM) with and without tetrodotoxin (1 μM), suggesting that the preceding enhanced activity is a prerequisite for the emergence of the Glu/GABA exchange process.

### Glu uptake and GABA release are coupled by changes in intracellular Na^+ ^level

**Figure 4:**
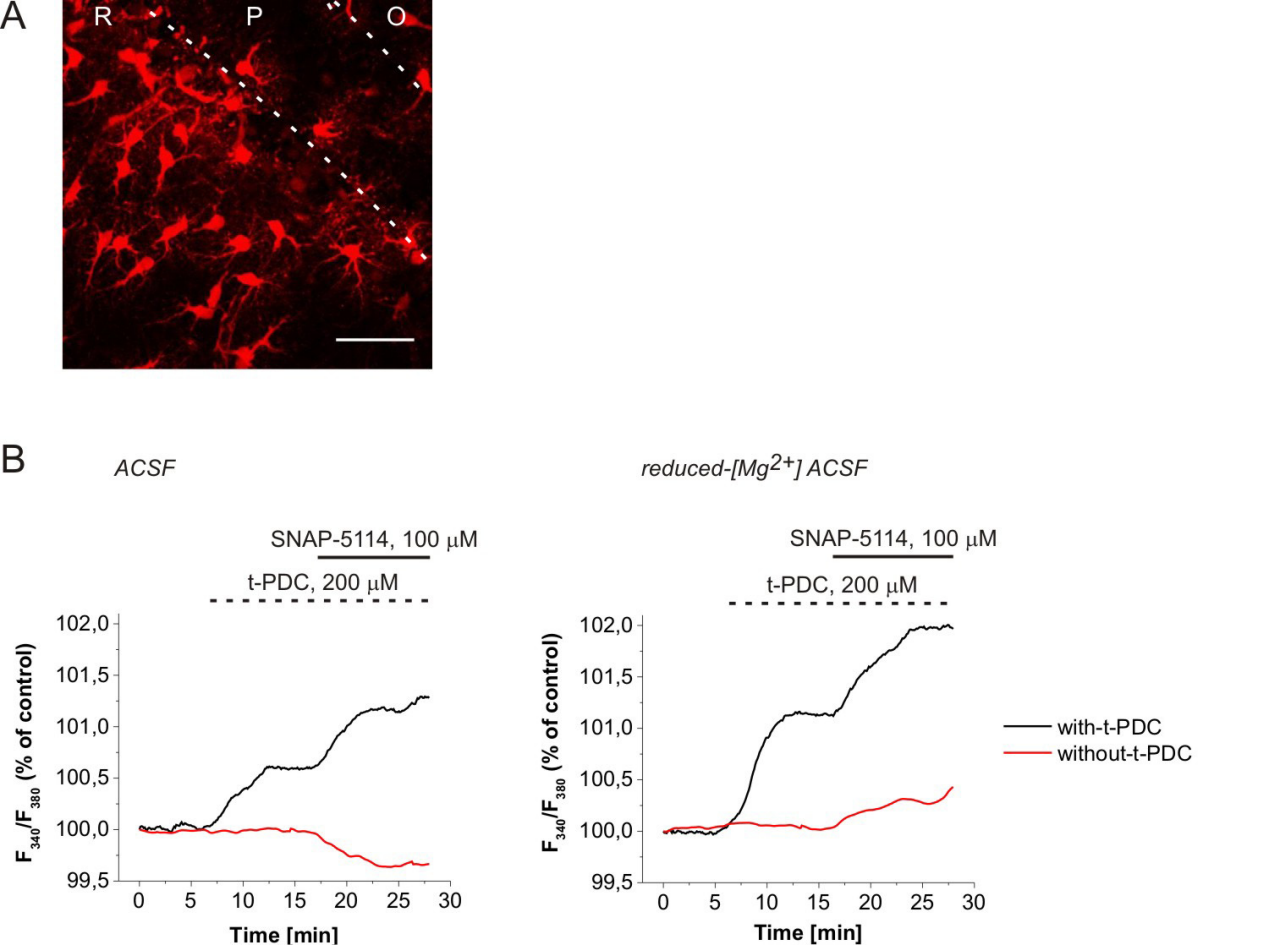
**Changes in glial [Na^+^] in response to EAAT activation and GAT-2/3 blockade**. **(A) **Confocal imaging reconstruction of protoplasmic astrocytes marked by sulforhodamine 101 (SR101) in the hippocampal CA1 region. R: str. radiatum, P: str. pyramidale, O: str. oriens. Scale bar = 50 μm. **(B) **Intracellular [Na^+^] in the soma of SR101 identified astrocytes measured by SBFI fluorescence during 100 μM SNAP-5114 applications with (black line) or without (red line) preceding activation of the Glu transporters by 200 μM t-PDC in normal ACSF (*left*) and [Mg^2+^] = 10 μM ACSF (right). Average of N = 16 cells/2 animals (normal ACSF) and N = 29 cells/3 animals ([Mg^2+^] = 10 μM).

Next we addressed how the Glu transporter activity can lead to GAT reversal. Both Glu and GABA transport gain their driving forces from the concentration gradient of Na^+ ^between intracellular and extracellular compartments [39]. The co-expression of EAAT2 and GAT-3 (Figure 1) raised the possibility that they share a common Na^+ ^pool; therefore, the activity of EAAT2 may directly affect the driving force of GAT-3. To investigate whether changes in intracellular Na^+ ^level is coupled to the Glu-evoked GABA release, we monitored the intracellular [Na^+^] in astrocytes in the CA1 str. radiatum and str. pyramidale of rat hippocampal slices. The dye sulforhodamine 101 (SR101) was used to mark EAAT expressing protoplasmic astrocytes [40] (Figure 4A) that express Glu transporters but not Glu receptors [41]. The glial [Na^+^] was monitored using the Na^+ ^ion-sensitive dye SBFI.

Under control conditions ([Mg^2+^] = 1,800 μM), application of SNAP-5114 (100 μM) decreased the glial [Na^+^], indicating that the glial GATs are taking up GABA and Na^+ ^(Figure 4B, left). Contrary, activation of the glial EAATs by the substrate t-PDC (200 μM) produced a significant increase of intracellular [Na^+^] as the result of an inward EAAT flux (Figure 4B, left). Subsequent blockade of the GAT-2/3 transporters by SNAP-5114 in the presence of t-PDC further increased the intracellular [Na^+^] (Figure 4B, left) indicating that GABA transporters work in the reversed mode following EAAT activation. Comparing these results with the electrophysiological measurements in ACSF on the GAT-2/3 mediated tonic current component in response to SNAP-5114 (Figure 2) and exogenous Glu suggest that although GABA can be released by the Glu/GABA exchange mechanism under control conditions as well, its amount is most probably not sufficient to activate the extrasynaptic GABA_A _receptors.

When the network activity was intensified using the [Mg^2+^] = 10 μM buffer, SNAP-5114 alone was able to increase the glial [Na^+^] (Figure 4B, r*ight*), indicating that glial GATs are reversed even without activating the EAATs by exogenously applied substrate in accordance with the electrophysiological results (Figure 2B). Further triggering the Glu/GABA exchange mechanism by 200 μM t-PDC resulted in a largely enhanced reverse uptake capacity for GAT-2/3 as indicated by the increased response to SNAP-5114 (Figure 4B, right).

It is worth emphasizing that the magnitude of the glial [Na^+^] changes in this experiment (Figure 4) underestimates the concentration changes in the vicinity of the transporters because the values were measured in the soma, far from the end feet where the most significant changes occur. As an example for [Na^+^] changes in a domain with the size comparable to the end feet of astrocytes, intracellular [Na^+^] may rise up to 100 mM in active dendritic spines of CA1 neurons [42].

### Alternative metabolic pathway provides releasable GABA in astrocytes

Electrophysiological studies outlined above clearly showed GAT-2/3 mediated GABA release from astrocytes. The source of glial GABA, however, remained undecided. GABA is synthesized mainly from Glu by the enzyme Glu decarboxylase. The majority of this pathway takes place in neuronal cells [43]; therefore, it is widely believed that astrocytes do not contain significant amounts of GABA. There is, however, an alternative metabolic pathway to GABA. The polyamine putrescine can be converted to GABA by monoamine oxidase [44] in neurons and glial cells as well [45]. Several lines of evidence suggest that this alternative pathway might play a neuroprotective role in multiple models of epilepsy [46-48]. We previously demonstrated by NMR measurements that Glu-induced GABA release is, indeed, independent of Glu decarboxylase [24]. Therefore, we sought to assess whether GABA synthesized from putrescine in glial cells may be the source of the release. Unfortunately, ^13^C or ^14^C labelled putrescine is unavailable currently. Consequently, we could not directly test whether the released GABA originates from putrescine.

Instead, we first opted to evaluate the potential of the putrescine-GABA pathway to produce significant amount of GABA by estimating the changes in the intracellular and extracellular concentrations of GABA and putrescine in the reduced-[Mg^2+^] environment. Since the ornithine-putrescine conversion represents the rate-limiting step in the polyamine synthesis [43], we also monitored the concentration of the putrescine precursor ornithine. Hippocampal slices were incubated in normal ACSF and in [Mg^2+^] = 1 μM ACSF for one hour. Following the incubation, the bathing solutions were removed and used to assess the extracellular concentrations. The slices were subject to a mild digestion procedure (*see *Methods) that is expected to selectively extract GABA from the cytosolic compartment and leave the vesicular stores intact. The digestion products were used to estimate the cytosolic concentrations. The putrescine, ornithine and GABA concentrations in both the slice extracts and the bathing solutions were determined by mass spectrometry. Under control conditions, cytosolic putrescine concentration was found to be 21.8 ± 1.6 pmol/mg wet tissue, while extracellular putrescine was measured as 12.2 ± 0.9 pmol/mg wet tissue, both in good agreement with data from other groups [49-51]. GABA concentration was calculated as approximately 1 mM in the cytosolic compartment and approximately 0.7 μM in the extracellular space, both in good agreement with previous values [52,53]. Although the absolute concentrations should be treated with considerable care due to the indirect sampling protocol, they suggest that we indeed sampled the appropriate pools.

**Figure 5:**
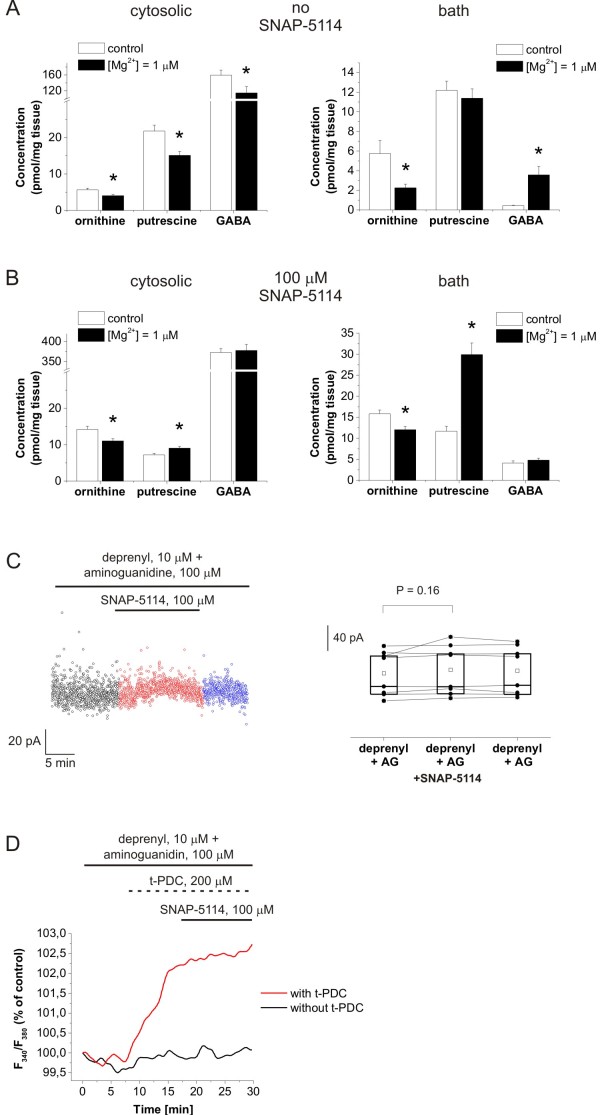
**GABA released from astrocytes is synthesized from putrescine. (A-B) **Concentration of ornithine, putrescine and GABA in hippocampal slice tissues and in the bathing medium following one hour incubation normal ACSF (control) or in [Mg^2+^] = 1 μM ACSF in the absence (A) and presence (B) of 100 μM SNAP-5114 as determined by LC-MS. Asterisks represent significant change (*P *< 0.05). **(C) **Holding currents in [Mg^2+^] = 1 μM buffer in the presence of the monoamino oxidase inhibitor, deprenyl (10 μM) and the diamino oxidase inhibitor, aminoguanidine (100 μM). Left, baseline currents plotted at 1 s intervals during control condition (black), SNAP-5114 application (red) and washout (blue); right, box-chart representation of GABA*ergic *baselines during control condition, SNAP-5114 application and washout. Box edges represent 25^th^, 50^th ^and 75^th ^percentile, open squares represent means, circles connected by lines represent paired individual baseline values. Average holding current in control: 74.8 ± 12.8 (N = 8 cells/6 animals). **(D) **Intracellular [Na^+^] in the soma of SR101 identified astrocytes measured by SBFI fluorescence during 100 μM SNAP-5114 applications with (black line) or without (red line) preceding activation of the Glu transporters by 200 μM t-PDC in [Mg^2+^] = 10 μM ACSF in the presence of deprenyl (10 μM) and aminoguanidine (100 μM) (average of N = 13 cells/2 animals).

We found that putrescine and ornithine concentrations were significantly decreased in the cytosolic pool (Figure 5A) in the reduced-[Mg^2+^] environment. Ornithine concentration was also decreased in the extracellular space when exposed to the reduced-[Mg^2+^] ACSF (Figure 5A). These data demonstrate the increased metabolic conversion of ornithine and putrescine. Cytosolic GABA concentration was also significantly decreased compared to the control condition. However, a remarkable increase was observed in the bath concentration of GABA (Figure 5A) in accordance with the tonic inhibition measurements (Figure 2). The increase in the bath concentration of GABA and the decrease in cytosolic [GABA] were both completely blocked by 100 μM SNAP-5114 (Figure 5B), demonstrating that GABA release was indeed mediated by GAT-2/3 transporters. When SNAP-5114 was present in the reduced-[Mg^2+^] buffer, both cytosolic and bath [putrescine] were increased (Figure 5B), suggesting that maintenance of the putrescine-GABA conversion requires the continual removal of the synthesized GABA. In the absence of GABA efflux the ornithine-derived putrescine is released to the extracellular space by the depolarization-induced polyamine secretory pathway [54,55]. The marked difference in the putrescine concentrations in the absence and presence of SNAP-5114 also signifies the correlation between the putrescine-GABA pathway and GAT-2/3 activity.

To confirm the contribution of the putrescine-derived glial GABA to the tonic inhibition of neurons, we investigated the tonic GABA*ergic *currents under blockade of the putrescine-GABA metabolic pathway using the monoamino oxidase inhibitor deprenyl in combination with the diamine oxidase inhibitor aminoguanidine [44]. In the [Mg^2+^] = 1 μM buffer that was used to investigate the Glu-induced GABA*ergic *currents, the presence of 10 μM deprenyl and 100 μM aminoguanidine eliminated the GAT-2/3 mediated tonic current component (Figure 5C).

Furthermore, the involvement of the putrescine-GABA pathway as the source of releasable glial GABA was also confirmed by measuring the glial [Na^+^] changes in response to the EAAT substrate t-PDC (200 μM) and the GAT blocker SNAP-5114 (100 μM) (Figure 5D) in the presence of 10 μM deprenyl and 100 μM aminoguanidine. Activation of the Glu transporters by t-PDC resulted in increased glial [Na^+^], however, this value could not be further increased by SNAP-5114 during either control conditions or enhanced network activity, suggesting that the releasable glial GABA is missing when the putrescine-GABA pathway is blocked (*cf*. Figure 4C). It is worth noting that the magnitude of the glial [Na^+^] increase in response to t-PDC in the presence of deprenyl and aminoguanidine (Figure 5D) was comparable to that measured in the presence of SNAP-5114 when the putrescine-GABA pathway was intact (Figure 4B), indicating that this [Na^+^] increase can be achieved when the GAT-2/3 transporters are not releasing the Na^+ ^to the extracellular space.

These data suggest that the putrescine-GABA metabolic pathway emerges during enhanced network activity and provides the glial GABA that contributes to the tonic inhibition of neurons.

### Glu uptake-induced GABA release from astrocytes reduces the duration of seizure-like events

**Figure 6:**
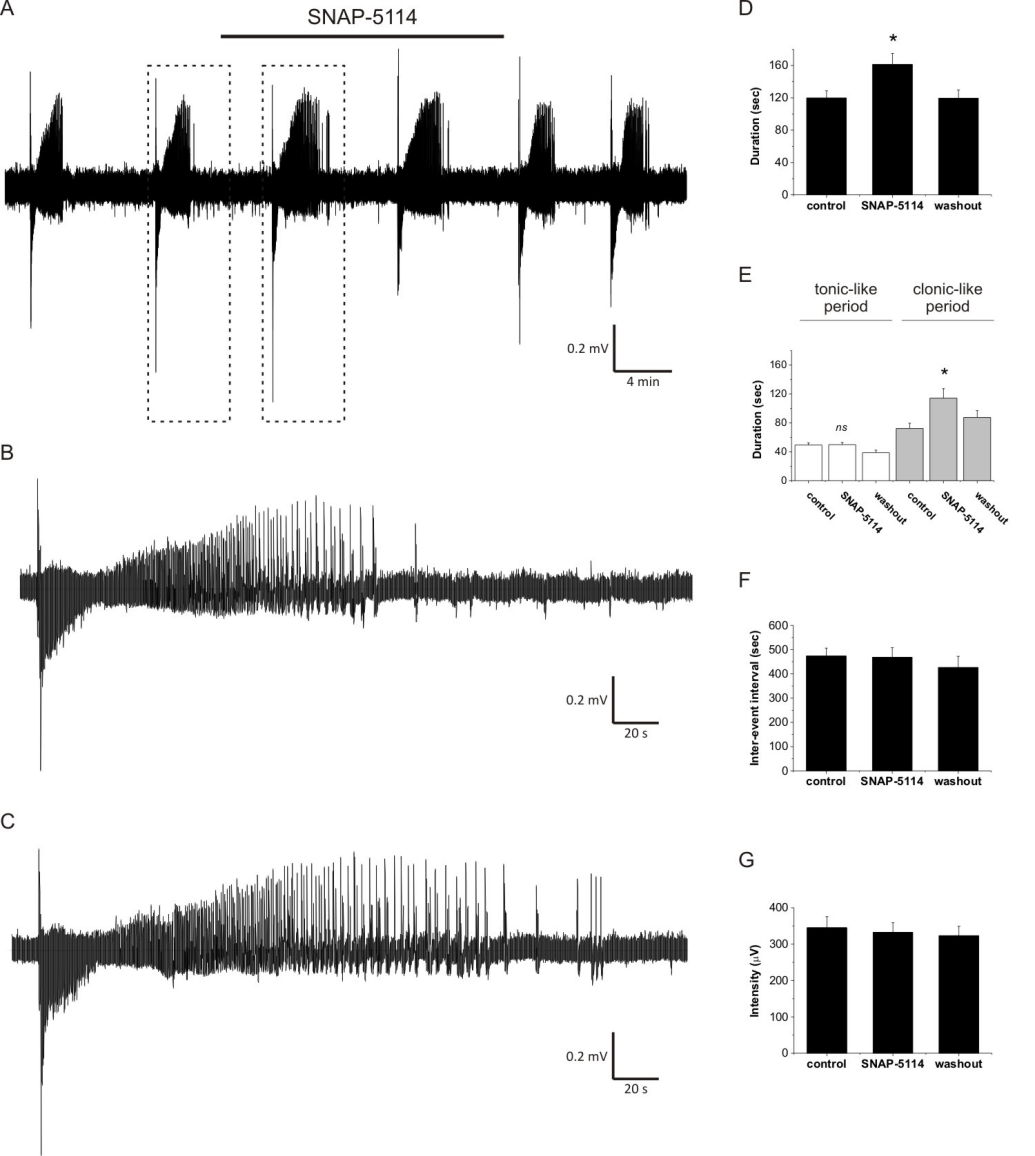
**Blockade of GAT-2/3 mediated tonic inhibition increase the duration of seizure-like events. (A) **Representative field potential recording of seizure-like events (SLEs) from the CA3 pyramidal layer of a hippocampal slice from a P11 rat. Bar shows SNAP-5114 (100 μM) application. **(B-C) **Comparison of SLEs (boxed in A) occurred during control condition (B) and SNAP-5114 application (C). **(D-G) **Average duration of SLEs (D), average duration of tonic- and clonic-like periods (E), average inter-SLE intervals (F) and average SLE intensity (G) from 13 slices exhibiting recurrent SLEs. Asterisks represent significant change compared to the control (*P *< 0.05), *ns *means not significant.

Since the Glu uptake-induced GABA release correlates with the network activity and the metabolic pathway yielding the glial GABA is also known to be induced during enhanced activity [48], it is reasonable to expect that the tonic current provided by astrocytes combats overexcitation in pathophysiological states characterized by sustained, enhanced network activity, such as epilepsy. To evaluate this possibility, we exposed freshly isolated, 400 μm entorhinal-hippocampal slices from P11-13 rats to nominally Mg^2+^-free ACSF with slightly elevated (5 mM) extracellular [K^+^]. In the low-[Mg^2+^] model of epilepsy [35-37], 73% of slices generated recurrent seizure-like events (SLEs) within 20 minutes from the exposure to low-[Mg^2+^]/elevated-[K^+^] ACSF. Following the appearance of at least two fully developed SLEs, we applied 100 μM SNAP-5114 to block the GAT-2/3 mediated tonic current component (Figure 6A-C). The effects of SNAP-5114 on the SLEs were quantified by measuring the total duration of the SLEs, the duration of the tonic- and clonic-like periods, the interval between two consecutive SLEs and the intensity of the SLEs (see Methods). Total duration of SLEs significantly increased from 119.9 ± 8.9 s (control) to 161.4 ± 13.6 s when the GAT-2/3 mediated tonic current component was blocked by SNAP-5114 (Figure 6D, P = 0.006, N = 13 slices/5 animals). The increase in duration specifically affected the clonic-like period (Figure 6E, 72.2 ± 7.3 s in control vs. 114.2 ± 13.2 s during SNAP-5114 application, *P *= 0.007), leaving the duration of the tonic-like period intact (Figure 6E, 49.3 ± 3.1 s in control vs. 49.9 ± 3.0 s during SNAP-5114 application, *P *= 0.84). The interval between SLEs and the SLE intensity were also unaffected by SNAP-5114 application (Figure 6F, G).

### The Glu/GABA exchange mechanism modulates electrophysiological properties under physiological conditions *in vivo*

By monitoring the glial [Na^+^] to determine whether glial GATs work in the normal or the reversed mode, we demonstrated that GAT-2/3 can be reversed by activating the glial Glu transporters under physiological conditions (Figure 4). However, the amount of the released GABA is most probably not sufficient to activate the extracellular GABA_A _receptors (Figure 2A). *In vitro*, the GABA provided by astrocytes can contribute to the tonic inhibition only when network activity is considerably enhanced. The network activity in an *in vitro *brain slice preparation, however, is inherently reduced compared to the *in vivo *intact brain [56]. To explore whether the Glu/GABA exchange mechanism can provide significant contribution to inhibitory actions *in vivo *under physiological conditions, we studied the effect of t-PDC and SNAP-5114 on the gamma range oscillation in the CA1 region of rats under ketamine/xylazine anesthesia.

The CA3 and CA1 gamma range (30 to 80 Hz) oscillations are hypothesized to share similar neuronal mechanisms under *in vivo *and *in vitro *conditions [57-59]. It is also well known that the hippocampal inhibitory neural network plays an important role in the gamma range oscillation [60]. It was also shown that gamma oscillation is coordinated by the intricate interplay between pyramidal cells and interneurons mostly by a dominant GABA*ergic *inhibitory input on pyramidal cells [61]. Importantly, under ketamine/xylazine anesthesia the CA1 region exhibits gamma range burst activity comparable to that during memory task performance [57], albeit in the low gamma range (30 to 50 Hz).

**Figure 7:**
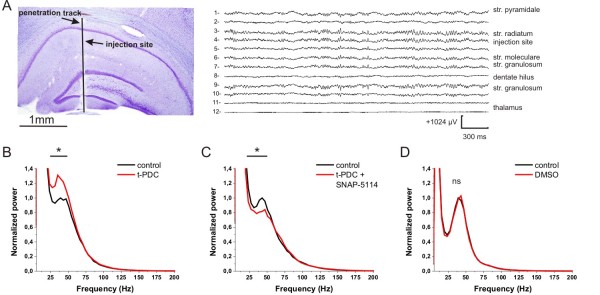
**Effect of t-PDC and SNAP-5114 on the gamma band activity in the CA1 region of rats under ketamine/xylazine anaesthesia**. **(A) **Left: Nissl stained section of the injector electrode penetration track. The vertical black line represents the track. Scale bar is 1 mm. Right: single sweep CSD activity from the str. pyramidale to the thalamus during high amplitude gamma activity in the hippocampus. Injection site was located between CSD channel 4 and 5. CSD values are in arbitrary units, time and amplitude calibration is 300 ms and 1024 μV respectively. **(B) **Effect of t-PDC (1 mM) on the CA1 str. radiatum CSD power compared to the pre-injection control. Black line with asterisk marks significant (*P *< 0.005) CSD power increase after t-PDC application. Power is normalized to the peak power of the control in the gamma (30 to 80 Hz) range. **(C) **Effect of simultaneous application of t-PDC (1 mM) and SNAP-5114 (1 mM) mixture on the CA1 str. radiatum CSD power compared to the pre-injection control. Black line with asterisk marks significant (*P *< 0.005) CSD power drop after t-PDC and SNAP-5114 application. **(D) **Effect of the saline solution containing 1% DMSO on the CA1 str. radiatum CSD power compared to the pre-injection control. No significant CSD power change was detected in the gamma range.

Topical injection (N = 3) of the EAAT substrate t-PDC (1 mM) to the str. radiatum of the CA1 (Figure 7A) resulted in the power increase of the current source density (CSD) in this region in the frequency range peaking around 31-35 Hz. This effect lasted for about four to eight minutes. Representative FFT power changes after t-PDC injection compared to the pre-injection period showed significant (*P *< 0.005) increase in the power of the gamma oscillation (Figure 7B). In contrast, t-PDC injected with 1 mM SNAP-5114 (N = 3) evoked a power suppression at gamma peak (Figure 7C). This suppression was also statistically significant in all injections in the CA1 str. radiatum, measured by the CSD FFT power spectrum. Injection of 1% dimethyl sulfoxide (DMSO) in saline solution (N = 3) that is used to dilute SNAP-5114 (and also added to the t-PDC solution to make the results strictly comparable) did not change the gamma power (*P *> 0.05) in the CA1 str. radiatum (Figure 7D).

## Discussion

The experiments in this paper establish, for the first time, a direct conversion of glutamat*ergic *excitation to GABA*ergic *inhibition by astrocytes. We showed that during enhanced network activity a previously unrecognized inhibitory mechanism emerges, by which the uptake of Glu is coupled to the subsequent reversal of the glial GABA transporters bringing about an elevation in the level of extracellular GABA and increasing the tonic inhibition of neurons. The presence of this Glu/GABA exchange mechanism was demonstrated by measuring the GAT-2/3 mediated tonic inhibitory current component of CA1 pyramidal cells and by monitoring the glial [Na^+^] under the blockade of glial Glu and GABA transporters in reduced-[Mg^2+^] conditions. We identified the polyamine putrescine as the source of the releasable glial GABA. Additionally, we revealed that the level of tonic inhibition provided by astrocytes is directly proportional to the network activity resulting in a tuneable, *in situ *negative feedback, which in turn counterbalances the excitation during recurrent seizure-like events in an *in vitro *epilepsy model. Furthermore, we provided evidence that the Glu uptake-induced glial GABA release occurs under physiological conditions *in vivo*, showing that increasing the extrasynaptic GABA*ergic *signalling by triggering the Glu/GABA exchange mechanism results in the modulation of gamma oscillation power.

### Proposed model of Glu induced GABA release

**Figure 8:**
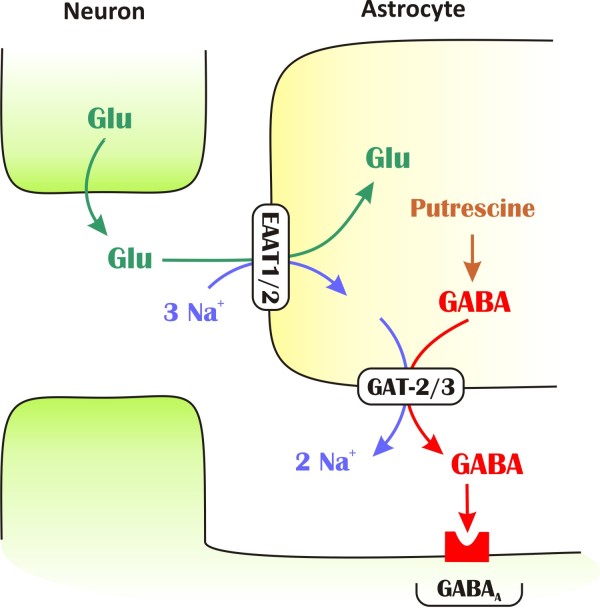
**Schematic representation of the mechanism underlying Glu/GABA exchange**. GAT-2/3 transporters are expressed on glial end feet surrounding glutamat*ergic *synapses and colocalize with EAAT2 transporters. Glial uptake of Glu is coupled to the release of GABA by a mechanism through which Na^+ ^that is cotransported with Glu drive the release of GABA synthesized from putrescine. The released GABA contributes to tonic inhibition of neurons.

To explain the current findings, we propose a model for the mechanism underlying extracellular Glu/GABA exchange by astrocytes (Figure 8). Importantly, we showed that the GAT-2/3 mediated tonic current component can be diminished by blocking glial Glu transporters (Figure 2C), demonstrating that GAT reversal is evoked directly by Glu uptake. Removal of Glu from the extracellular space is coupled [39] to co-transport of 3 Na^+^/1 H^+ ^and counter-transport of 1 K^+^, resulting in subsequent disruption of the resting electrochemical potential. Because GABA transport is also driven by the Na^+ ^gradient, the increased intracellular [Na^+^] may be capable of reversing GABA transporters. Both theoretical and experimental studies suggest that GABA transporters operate close to their equilibrium potential [18,62,63]; therefore, even small changes in the concentration of the underlying substrates (Na^+^, Cl^- ^or GABA) may initiate the reversed mode. Indeed, we found that activation of Glu transporters significantly increases the intracellular [Na^+^] even in the soma of astrocytes (Figure 4). Following EAAT activation, blockade of GAT-2/3 further elevates glial [Na^+^] demonstrating that glial GABA transporters are reversed. Importantly, the [Na^+^] changes in the local environment of the transporters are expected to be more substantial. Taking into account the presence of GAT-2/3 on astrocytic end feet ensheathing glutamat*ergic *synapses [25,26] and the colocalization of EAAT2 and GAT-2/3 (Figure 1), the glial Glu and GABA transporters may share a common domain in the astrocytic end feet. Therefore, the Na^+ ^influx through EAATs may robustly change the driving force of GATs and can lead to transporter reversal.

According to our model, the released GABA activates the extrasynaptic GABA receptors and contributes to the tonic inhibition of neurons. This transporter-mediated molecular interplay represents a direct link between inhibitory and excitatory neurotransmission and functions as an adjustable negative feedback that may combat intense excitation in epilepsy. Indeed, we found that in the low-[Mg^2+^] model of epilepsy, blockade of the GABA release through GAT-2/3 transporters significantly increases the duration of seizure-like events (Figure 6), signifying the role of Glu/GABA exchange process in neuroprotection. Moreover, beyond the primary benefit of turning excitation into inhibition, the Glu/GABA exchange also contributes to the recovery of the transmembrane Na^+ ^gradient without using ATP thereby saving energy while protecting the neurons. Our *in vivo *findings of increased power in the gamma range due to t-PDC application and its elimination by SNAP-5114 also strengthen the hypothesis that Glu induced GABA release can play an important role in the hippocampal excitability.

### Occurrence of the Glu/GABA exchange mechanism under *in vitro *conditions

It is an important issue under what circumstances the Glu/GABA exchange appears. Under *in vitro *conditions we observed GAT-2/3 mediated tonic current component only in low-[Mg^2+^] environment. Although glial [Na^+^] measurements suggest that the GAT-2/3 transporters can be reversed by activation of the Glu transporters under normal, physiological activity (Figure 4B, left), blockade of GAT-2/3 did not change the tonic inhibition of CA1 pyramidal neurons (Figure 2A) under the same conditions. The apparent discrepancy between the measurements of glial [Na^+^] level and of tonic inhibition may denote that the GABA released through GAT-2/3 is either taken up before reaching the extracellular GABA_A _receptors or its concentration is not sufficient to activate them. Since GAT-2/3 are localized on glial processes facing the extrasynaptic space [25,26] where the GABA_A _receptors responsible for the tonic inhibition reside [64], the synaptically localized GAT-1 transporters cannot take up the released GABA. Therefore, having no major route to remove GABA from the extrasynaptic space, it is more likely that under control conditions the released amount of GABA is not sufficient to activate extrasynaptic GABA_A _receptors.

Enhancing the network activity by lowering the extracellular [Mg^2+^] turns on the Glu/GABA exchange process as monitored by both glial [Na^+^] measurements (Figure 4B, right) and GABA*ergic *tonic inhibition (Figures 2B and 3A). Lowered [Mg^2+^] and increased network activity may lead to diverse physiological actions. As some examples, low Mg^2+ ^level can open up TRPM7 channels resulting in Na^+ ^influx [65] and subsequent depolarization of neurons. The increased network activity can also initiate overexpression of glial Glu and GABA transporters [66-68] that may enhance the magnitude of the Glu/GABA exchange process. Moreover, reversal of glial GABA transporters can also be facilitated by increased glial GABA production in response to the elevated extracellular [Glu] [69].

Glial GABA production can also be enhanced by activation of the putrescine-GABA pathway. Although the source of glial GABA is poorly understood, it has been demonstrated that GABA is indeed available in astrocytes [70-72]. Apart from the major source of GABA (forming from Glu by glutamate decarboxylase in neurons), an alternative pathway does exist in the brain by which GABA is formed by monoacetylation of the polyamine putrescine in both neurons and glial cells [73]. Although the capacity of this pathway is modest, it can produce detectable levels of GABA in astrocytes [74]. Importantly, this pool of GABA is inducible. The formation of GABA from putrescine is markedly increased in the epileptic mouse strain DBA/2J [48]. Also, long-lasting increase in putrescine level was demonstrated following kainate-induced seizure activity in rat brain *in vivo*, particularly in the hippocampus [46]. Moreover, putrescine production can also be induced by glutamine [75]; therefore, it can be directly coupled to the uptake of Glu that is converted to glutamine by astrocytes. The activity induced formation of GABA from putrescine, therefore, is a good candidate to explain the source of the released GABA and may also enlighten why putrescine overproduction offers neuroprotection by significantly increasing seizure threshold [47].

In conclusion, among the various physiological changes in response to the low-[Mg^2+^] environment, we consider the activity-induced enhancement of the putrescine-GABA synthetic pathway as the major determinant of the appearance of the Glu/GABA exchange mechanism, because its blockade eliminated the GAT-2/3 mediated component of both the tonic inhibition (Figure 5B) and the glial [Na^+^] increase (Figure 5C).

### Occurrence of the Glu/GABA exchange mechanism under *in vivo *conditions

Either the activation of the putrescine-GABA pathway or some other mechanisms underlie the emergence of the Glu/GABA exchange, the GAT-2/3 mediated tonic inhibitory component could only be detected during intensified network activity under low-[Mg^2+^] conditions *in vitro*. The network activity in an *in vitro *brain slice preparation, however, is inherently reduced when compared to the *in vivo *intact brain [56]. It is, therefore, feasible that the intrinsically higher *in vivo *network activity may provide sufficient drive to trigger the Glu/GABA exchange mechanism. Indeed, the gamma range oscillation that is known to be coupled to the GABA*ergic *inhibitory input on pyramidal cells [61] has been modulated by the Glu transporter substrate under *in vivo *physiological conditions. Although the application of a Glu transporter substrate/inhibitor may affect the network dynamics in several different ways, it is important that the modulatory effect of t-PDC could be fully reversed by SNAP-5114. If the gamma power increase was produced by a rise in the extracellular Glu in response to t-PDC application, the resulting excitation cannot be eliminated by the blockade of the glial GABA transporters that are working in the standard, outside-in direction, due to the minor contribution of GAT-2/3 to the total GABA uptake capacity. The inversion of the t-PDC induced gamma power modulation by SNAP-5114, therefore, suggests that they are acting on different targets of the same mechanism.

### Potential therapeutic implications

The magnitude of tonic inhibition provided by glial Glu/GABA exchange is proportional to the degree of network activity, thus it is more prominent in excited states. From the pharmacological point of view, treatments that target a mechanism up-regulated *in situ *in pathological conditions may represent an ideal strategy for drug development.

The coupling between substrate activation of EAATs and subsequent GABA release can well explain some previous observations. The mechanism implies that transportable and non-transportable inhibitors of Glu uptake should have differential effects on neuronal viability. Indeed, this distinction was observed in several cases. In contrast to the non-transportable blocker DHK, the substrate t-PDC did not evoke neuronal damage *in vivo *even in very high concentrations (25 to 100 mM), despite the fact that extracellular [Glu] elevation was similar [76] or even higher [77] after t-PDC application than following DHK treatment. Also, *in vivo *administration of TBOA in the hippocampus was demonstrated to induce neuronal damage in the CA1 and the dentate gyrus, while t-PDC application did not produce cell death [78].

Overexpression of glial Glu and GABA transporters may significantly increase the magnitude of the Glu-induced GABA release by increasing both the Na^+ ^influx through EAATs and the amount of GABA released through GATs. The antiepileptic drugs clobazam and levetiracetam have been shown to up-regulate GAT-3 expression in the hippocampus [66,67]. The marked increase of GAT-3 expression in astrocytes was also demonstrated in the hippocampi of patients with temporal lobe epilepsy [68]. The dysfunction of the mechanism may also lie behind the impairment of the cross-talk between excitatory and inhibitory transport processes in temporal lobe epilepsy [79].

## Conclusions

This study, together with our previous work, demonstrates the existence of a previously unrecognized adaptive neuroprotective mechanism, by which astrocytes offer an *in situ*, tuneable negative feedback on neurons. In acute hippocampal slices, the mechanism can be observed only during enhanced network activity evoked by low-[Mg^2+^] condition. However, occurrence of the Glu/GABA exchange mechanism *in vivo *suggests that the Glu/GABA exchange can be operational under physiological conditions in the intact brain. We envision that the discovery of the conversion of glutamat*ergic *excitation to tonic inhibition will deepen our understanding of how physiological network activity can be regulated in the brain and may open up new possibilities for the treatments of pathological conditions, such as epilepsy or ischemia.

## Methods

Animals were kept and used in accordance with the European Council Directive of 24 November 1986 (86/609/EEC) and the Hungarian Animal Act, 1998 and associated local guidelines. All efforts were made to reduce animal suffering and the number of animals used.

### Buffers

Buffers contained in mM ACSF: 129 NaCl, 3 KCl, 1.6 CaCl_2_, 1.8 MgSO_4_, 1.25 NaH_2_PO_4_, 21 NaHCO_3_, 10 glucose (pH 7.4); nominally Mg^2+^-free ACSF was prepared as control ACSF with no added Mg^2+ ^(based on the Mg^2+ ^contamination of the Ca^2+ ^salts, we estimated the Mg^2+ ^concentration of this buffer to be approximately 1 μM).

### Slice preparation

Transverse, 300 μm thick hippocampal-entorhinal slices from 10- to 18-day-old Wistar rats (Toxicoop, Budapest, Hungary) were prepared in modified ACSF (75 mM sucrose, 87 mM NaCl, 2.5 mM KCl, 1.25 mM NaH_2_PO_4_, 7 mM MgSO_4_, 0.5 mM CaCl_2_, 25 mM NaHCO_3_, 25 mM glucose, continuously bubbled with 95% O_2 _+ 5% CO_2 _gas mixture) at 4°C, as described before [80]. In most of the experiments (158 out of 163), animals between P11-15 were used, while in measurements of recurrent seizure like activity (SLE) the age range was further reduced to P11-13 since they are more susceptible to developing seizures [35,80]. Slices were incubated in an interface-type chamber that was continuously circulated with ACSF for one hour at 37°C (followed by incubation at room temperature) before performing the experiments. In lowered [Mg^2+^] experiments slices were incubated in ACSF containing approximately 100 μM. In experiments where the diamine oxidase inhibitor, aminoguanidine, was applied, aminoguanidine was added to the ACSF before incubating the slices in the interface chamber in order to effectively block the putrescine-GABA synthetic route.

### *In vitro *electrophysiology

Electrophysiological recordings were performed either at room temperature or at 31°C. Signals were recorded with Multiclamp700A amplifiers (Axon Instruments, Foster City, CA, USA), low-pass filtered at 2 kHz and digitized at 10 kHz (Digidata1320A, Axon Instruments). For single cell recording CA1 pyramidal cells were identified visually. Pipettes (5 to 9 MΩ) were filled with a solution containing (in mM) 130 CsMeSO_3_, 10 NaCl, 0.05 CaCl_2_, 2 ATP (magnesium salt), 1 EGTA and 10 HEPES (pH set to 7.3 with 1N CsOH). To suppress escape action currents 5 mM QX 314 (Tocris, Bristol, UK) was added. Cells were voltage-clamped at 0 mV (corrected for a calculated junction potential of +15 mV) to record GABA*ergic *(outward) currents. Input resistance was 171 ± 65 MΩ. If signs of seal deterioration or cell closure occurred (> 20% change in the access resistance) the recordings were discarded. Synaptic recordings were made for 10 to 25 minutes in control conditions following 10 to 20 minutes of 100 μMSNAP-5114 application and 10 to 30 minutes washout.

In experiments where local Glu release at CA1 pyramidal cells was evoked, 100 μs, 500 μA stimuli were applied to the Schaffer collaterals by a bipolar Tungsten electrode at 15 s intervals. Experiments were discarded if stimulation of Schaffer collaterals did not evoke Glu*ergic *current in the CA1 pyramidal cell voltage clamped at -45 mV. Sweeps were recorded for 9 s following the stimulus and the ranges between 1.5 to 8 s were used to analyze spontaneous IPSCs and to determine the baseline in order to exclude evoked responses.

**Figure 9:**
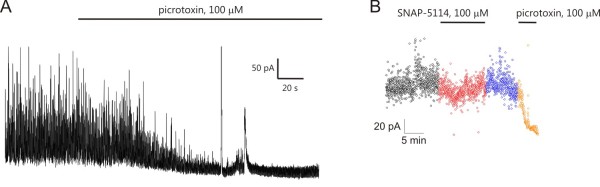
**Validation of phasic and tonic currents recorded from CA1 pyramidal neurons at 0 mV holding potential as GABAergic currents. (A) **Voltage-clamp recording showing the elimination of both GABA*ergic *IPSCs and GABA*ergic *tonic current in the presence of 100 μM picrotoxin in ACSF containing 30 μM Mg^2+^. **(B) **Holding current plotted at 1 s intervals during control condition (black), SNAP-5114 application (red), washout (blue) and picrotoxin application (brown) in the same experiment.

Synaptic currents were identified as GABA*ergic *inhibitory postsynaptic currents and baseline currents were validated as a measure of tonic GABA*ergic *currents by adding the GABA_A _antagonist picrotoxin (100 μM) at the end of 43 of 154 experiments (Figure 9). Picrotoxin sensitive tonic current was found to be 53.1 ± 10.7 pA, 57.1 ± 8.4 pA and 63.4 ± 10.7 pA at [Mg^2+^] = 1, 10 and 30 μM, respectively.

Holding currents were determined according to Glykys *et al. *[81]. All-point histograms were plotted for each sweep (in episodic recording mode when the Schaffer collaterals were stimulated) or for each 20 s period of experimental traces (in gap-free recording mode). A Gaussian was fitted to the unskewed part of the histogram and the position of the center of the fitted Gaussian was used as the holding current. Values during SLEs were not included in data evaluation. Experiments were discarded if the holding current continuously shifted to either a negative or positive direction during both SNAP-5114 or Glu application and washout, except when the shift was clearly linear, in which case the holding current values were detrended. Spontaneous IPSCs were analyzed by the MiniAnalysis software (Synaptosoft, Decatur, GA, USA) using 10 pA as amplitude threshold. sIPSCs with event frequency values greater than 300 Hz were excluded from histogram plots to avoid duplicate sIPSC detection.

Epileptiform activity was induced in 400 μm thick hippocampal slices by switching the perfusing solution to ACSF with no added Mg^2+ ^ions and [K^+^] raised to 5 mM. Field potential recordings were performed with glass microelectrodes (3 to 5 MΩ) inserted into the CA3 stratum pyramidale. Slices were discarded if SLE did not appear in 20 minutes starting from the exposure to low-[Mg^2+^]/elevated-[K^+^] ACSF. Being not fully developed, the first SLEs were always discarded from data evaluation. Recordings were analyzed after high-pass filtering at 1 Hz. Tonic-to-clonic transitions were identified by the first reappearance of secondary discharges. SLE intensity was calculated as the standard deviation (SD) of the field potential trace containing the whole SLE, normalized to the SD of the preceding period.

### *In vivo *electrophysiology

Rats (N = 6) weighing 250 to 350 g were used for the *in vivo *experiments. All procedures followed NIH Guidelines for the Use of Laboratory Animals. In each animal, pressure injections of saline (0.9% w/v) + DMSO (1%), t-PDC (1 mM, in saline containing 1% DMSO) and t-PDC (1 mM) + SNAP-5114 (1 mM) (in saline containing 1% DMSO) were made into the CA1 area of the hippocampus, in approximate stereotactic coordinates AP 3.0, ML 2.0 and DV 3.0 mm [82]. CA1 was also identified by the electrophysiological recordings, guided by the appearance of the large amplitude gamma oscillations (30 to 80 Hz) in local field potential (LFP) recordings. At the end of the experiments the animals were sacrificed, the brains were removed and histology confirmed the localization of the electrodes in Nissl stained sections [83].

Anaesthesia was induced by the intramuscular injection of a mixture of ketamine and xylazine (100 mg/kg and 10 mg/kg, respectively) and maintained by repeated (approximately every 30 minutes) intramuscular administration of the same substance. Body temperature was kept at 37°C with a heating pad. The head was held by a rat adaptor affixed to a stereotaxic frame (David Kopf Instruments, Tujunga, CA, USA). Midline incision was made on the scalp exposing the skull, followed by retraction of the skin and craniotomy to expose a part of the left hemisphere. The dura was left intact and room temperature saline solution was used to prevent desiccation.

A 23 channel laminar multi-electrode equipped with two inner cannulae (40 μm diameter glass capillaries) was used to record field potentials and to deliver the testing solutions. The injector electrode was lowered through the intact dura to target the CA1 region using a microdrive. Interelectrode spacing was 150 μm, electrode site diameter was 25 μm, shaft diameter was 350 μm, the drug delivery site was located between contacts 5 and 6 from the top (corresponding to current source density channel 4 and 5 due to the fact that during current source density computation the first and last channel is lost). The cannulae were attached to two calibrated micrometer driven 5 μl Hamilton syringes (Hamilton Company, Reno, NV, USA) via a 250 μm inner diameter Tygon tube (Saint-Gobain, Akron, OH, USA). Separate cannulae were carefully forward- and back-filled with the testing solutions to avoid air bubbles in the tubes.

LFP (0.03 Hz to 5,000 Hz) was recorded from each of the contacts, sampled at 20 kHz/channel rate with 16 bit precision (LabView, National Instruments, Austin, TX, USA) and stored on a hard drive for off-line analysis. Current source density (CSD) analysis identifies synaptic/transmembrane generators of LFP, using high-resolution maps of simultaneously recorded field potentials obtained across a laminated neural structure. Inhomogeneous conductivity was not taken into account, second spatial derivative was calculated by the nearest neighbour method, and high spatial frequency noise was reduced by Hamming-window smoothing [84,85]. Artefact free single sweep CSD epochs (256 ms long) were averaged (N = 1,000) in the frequency domain using FFT to obtain the power spectrum for all of the conditions. The CSD power spectra in the str. radiatum of the CA1 before and after the testing solution pressure injection (500 nl) were compared using t-test, significance level was set to *P *= 0.005 (t = 2.57).

### Double immunolabeling of GAT-3 and EAAT2

In the first step, an affinity-purified polyclonal antiserum (rabbit anti-GAT-3, cat# AB1574, Chemicon, Temecula, CA, USA) was applied to label GAT-3. The epitope for the anti-GAT-3 antiserum was the C-terminus of rat GAT-3 (aa 607 to 627) coupled to keyhole limpet hemocyanin. No cross-reactivity to the C-termini of other transmitter transporters was detected for the anti-GAT-3 antiserum (see the manufacturer's technical data sheet). Subsequently, sections were immunolabeled for EAAT2 using a monoclonal mouse anti-EAAT2 antiserum (mouse anti-EAAT2, cat# ab77039, Abcam, Cambridge, UK).

The immunostaining was performed by using Alexa594 labeled secondary antibody (cat# A21207, Life Technologies, Grand Island, NY, USA) for GAT-3 followed by FITC-tyramide fluorescent amplification immunocytochemistry for EAAT-2. Briefly, free-floating brain sections were pretreated in phosphate buffer (pH = 7.4; PB) containing 0.5% Triton X-100 and 3% bovine serum albumin for one hour. Then, they were incubated with primary antibody against GAT-3 (1:50) in PB containing 0.5% Triton X-100, 3% bovine serum albumin, and 0.1% sodium azide for 48 hours at room temperature. Sections were then incubated in Alexa 594 donkey anti-rabbit secondary antibody (1:400) for two hours. After washing, the sections were incubated overnight in the anti-EAAT-2 antibody followed by incubation in biotin-conjugated donkey anti-mouse secondary antibody at 1:1,000 (Jackson ImmunoResearch, West Grove, PA, USA) for two hours, followed by incubation in avidinbiotin-horseradish peroxidase complex (ABC) at 1:500 (Vectastain ABC Elite kit, Vector, Burlingame, CA, USA) for two hours. Then, sections were treated with fluorescein isothiocyanate (FITC)-tyramide (1:8,000) and 0.003% H_2_O_2 _in Tris-HCl buffer (0.05 M, pH 8.2) for eight minutes, washed, mounted on positively charged slides (Superfrost Plus, Fisher Scientific, Fair Lawn, NJ, USA), and cover-slipped in antifade medium (Prolong Antifade Kit, Life Technologies, Grand Island, NY, USA).

Sections were examined by using an Olympus BX60 light microscope (Olympus Corporation, Tokyo, Japan) also equipped with fluorescent epi-illumination. Images were captured at 2,048 × 2,048 pixel resolution with a SPOT Xplorer digital CCD camera (Diagnostic Instruments, Sterling Heights, MI, USA) using 4 to 40x objectives. Confocal images were acquired at 1,024 × 1,024 pixel resolution with a Nikon Eclipse E800 confocal microscope (Nikon Corporation, Tokyo, Japan) equipped with a BioRad Radiance 2100 Laser Scanning System (Bio-Rad Laboratories, Hercules, CA, USA) using 20 to 60x objectives at optical thicknesses of 1 to 5 μm. Contrast and sharpness of the images were adjusted by using the levels and sharpness commands in Adobe Photoshop CS 8.0 (Adobe Systems, San Jose, CA, USA). Full resolution was maintained until the photomicrographs were cropped and assembled for printing, at which point images were adjusted to a resolution of 300 dpi.

### [Na+] monitoring in astrocytes

We used 250 μm hippocampal slices loaded with sodium-binding benzofuranisophthalate (35 μM, Molecular Probes) in the presence of 0.07% Pluronic-127 (Molecular Probes) in ACSF for one hour at 37°C. Pluronic-127 was dissolved in DMSO. Astrocytes were marked [40] by applying 1 μM sulforhodamin 101 (SR101) for 10 minutes at 37°C. To validate the morphology of SR101-labelled cells, slices were imaged with an Olympus FV300 laser scanning confocal microscope system. Excitation was performed at 543 nm, emitted light was filtered with a 560 to 600 nm bandpass filter. Images were obtained by summation of optical sections taken in the Z-axis, using ImageJ (NIH) software.

Conventional, wide-field fluorescence imaging was performed using a digital imaging system (Olympus BX-FLA) attached to an upright microscope (Olympus BX50WI, 40x water immersion objective) and a CCD camera as sensor (Princeton Micromax, Princeton Instruments, Trenton, NJ, USA). Fluorescence excitation wavelengths were selected by using a high-speed wavelength switcher (Sutter Lambda DG-4, Sutter Instrument, Novato, CA, USA). Image acquisition at 0.1 Hz and time series were computer-controlled using the software Metafluor.

For wide-field imaging with SBFI-AM at 37°C, fluorescence signals from astrocytes previously identified by sulforhodamine 101 were collected at 525 nm (45 nm bandwidth) after alternate excitation at 340 nm and at 380 nm, and the fluorescence ratio (F_340_/F_380_) was calculated. At the end of some experiments an *in situ *calibration was performed [86] by permeabilizing cells for Na^+ ^using gramicidin (6 μg/ml, Sigma, Sigma-Aldrich, St. Louis, MO, USA) and monensin (10 μM, Sigma) with simultaneous inhibition of the Na^+^/K^+^-ATPase with ouabain (1 mM, Tocris) in a buffer containing 1.23 mM KH_2_PO_4_, 1.8 mM MgSO_4_, 1.6 mM CaCl_2_, 21 mM KHCO_3 _and 10 mM glucose. Slices were then sequentially perfused with solutions containing 0, 5, 10, 20 and 50 mM Na^+^, keeping (NaCl + choline-chloride) concentration at 150 mM. A five-point calibration curve was computed for each selected cell in the field of view and used to convert fluorescence ratio values into Na^+ ^concentrations. Baseline [Na^+^] was 4.3 ± 0.05 mM in average of 29 cells, K_d _for SBFI was found to be 7.52 ± 0.82 mM.

### Mass spectrometry

After pre-incubation for three to five hours in an interface-type incubation chamber, seven 300 μm hippocampal slices were placed on the bottom of a well in a 24-well plate. Following one-hour incubation in 300 μl of either normal ACSF or [Mg^2+^] = 1 μM ACSF, the bath solution was removed and used as a measure of the extracellular environment. The slices were transferred to a micro tube. The remaining small amount of buffer was removed from the tube and the slices were weighted to obtain the wet tissue weight. In order to circumvent the high background in GABA concentration measurements due to the extreme level of GABA in neurotransmitter vesicles, the slices were then subject to a mild digestion procedure by freezing and thawing three times for 10 minutes per cycle. This protocol is supposed to selectively extract the cytosolic compartment leaving the neurotransmitter vesicles intact. Using [^3^H] GABA to selectively label the cytosolic or vesicular pools [87], we verified that this procedure selectively extracts the cytosolic compartment. After the freezing-thawing cycles, the fragmented membrane homogenate was centrifuged at 61,000 g for 20 minutes and the supernatant was used as a measure of the cytosolic environment. The GABA concentration in these samples was found to be approximately 1 mM, further confirming its cytosolic origin.

The samples were separated prior to mass spectrometric analysis by a Perkin Elmer Series 200 Micro HPLC system (Norwalk, CT, USA) consisting of a binary pump, an autosampler and a column oven compartment. The modified method of Eckstein [88] was used where the mobile phase A was 1% formic acid and 0.5% heptafluoro-butiric acid (HFBA) in water and mobile phase B was 1% formic acid and 0.5% HFBA in acetonitrile. The column used was a Phenomenex Synergy Hydro-RP 80A (Torrance, CA, USA) (150 × 3 mm, 4 μm). Oven temperature was 45°C. The initial mobile phase composition was 100% A for 3.5 minutes then a linear gradient was applied for 5.5 minutes to 90% B. This was maintained for 1.0 minute and a quick linear gradient back to 100% A for 0.5 minute was followed by a 3.5 minutes equilibrium period. The overall run time was 14.0 minutes. The flow rate of the mobile phase was 500 μl/minute.

For quantitative analysis of compounds of interest an AB Sciex 3200 Qtrap tandem mass spectrometer (AB Sciex, Foster City, CA, USA) was used. The instrument was run in positive electrospray multiple reaction monitoring (MRM) mode. The source conditions were: curtain gas 20 l/minute, GS1 and GS2 50 and 40 l/minute, respectively, temperature of the drying gas 500°C, spray voltage 5000 V and the declustering potential was 20 V. The MRM transitions and collision energies were (Q1/Q3, CE) GABA 104/87, 20 ornithine 133/70, 20 and putrescine 89/72, 25. The dwell time was 100 ms for all transitions. Due to the different expected concentrations of the target compounds in cytosolic and bath samples, different calibration points were used. A five-point calibration curve was used in the range of 1 to 100 μM and only 10 μl of samples were injected for cytosolic samples. For bath samples, a seven-point calibration curve in range of 0.05 to 10 μM was used with injection volume of 30 μl. The built-in quantitation module of Analyst 1.5.1 software (Framingham, MA, USA) was used for the quantitation.

The concentrations of the analytes were finally calculated as pmol/mg wet tissue for both the cytosolic and the bath samples. Molar GABA concentrations were estimated by converting the pmol/mg values assuming 0.8 g/ml tissue density and 0.17 as the ratio of extracellular/total volume.

### Data evaluation

Unless stated otherwise data are expressed as means ± S.E.M. and were analyzed using Student's paired *t*-test or one-way analysis of variances with Bonferroni *post hoc *tests (OriginPro 8.0, OriginLab Coporation, Northampton, MA, USA). A value of *P *< 0.05 was considered significant.

## Abbreviations

ACSF: artificial cerebrospinal fluid; AMPA: 2-amino-3-(5-methyl-3-oxo-1,2- oxazol-4-yl)propanoic acid; AP5: (2*R*)-amino-5-phosphonovaleric acid; CNQX: 6-cyano-7-nitroquinoxaline-2,3-dione; CSD: current source density; DHK: dihydrokainate; DMSO: dimethyl sulfoxide; EAAT: excitatory amino acid (glutamate) transporter; EGTA**: **ethylene glycol tetraacetic acid**; **GABA**: **γ-aminobutyric acid; GAT: GABA transporter; Glu: glutamate; HEPES: 4-(2-hydroxyethyl)-1-piperazineethanesulfonic acid; IPSC: inhibitory postsynaptic current; LFP: local field potential; NMDA: N-methyl-D-aspartate; SBFI: sodium-binding benzofuranisophthalate; SLE: seizure-like event; SNAP-5114: 1-[2-[tris(4-methoxyphenyl)methoxy]ethyl]-(S)-3-piperidinecarboxylic acid; SR101: sulforhodamine 101; t-PDC: L-trans-pyrrolidine-2,4-dicarboxylic acid.

## Competing interests

The authors declare that they have no competing interests.

## Authors' contributions

LH participated in experimental design, carried out glial [Na^+^] measurements, participated in the *in vitro *electrophysiological studies and drafted the manuscript. GN and OK participated in the *in vitro *electrophysiological studies. ÁD performed the immunoassays. PS carried out MS measurements. RF carried out the *in vivo *electrophysiological studies. IU designed the *in vivo *electrophysiological studies and analyzed the results. BPS participated in experiment design. MP participated in immunoassay experimental design. JK participated in experimental design, coordinated the study and helped to draft the manuscript. All authors read and approved the final manuscript.
